# Ivermectin Prescription Fill Rates Among U.S. Military Members During the Coronavirus Disease 2019 (COVID-19) Pandemic

**Published:** 2024-01-20

**Authors:** Shawn S. Clausen, Jessica H. Murray, Shauna L. Stahlman

**Affiliations:** 1 Uniformed Services University of the Health Sciences; 2 Epidemiology and Analysis Branch, Armed Forces Health Surveillance Division, Defense Health Agency

## Abstract

This report describes ivermectin prescription fill rates among U.S. active component service members (ACSM) over time during the early phases of the COVID-19 pandemic. Information about the unsubstantiated benefits of ivermectin for coronavirus 2019 (COVID-19) prevention and treatment was widely available online early in the COVID-19 pandemic. Ivermectin prescription fill rates increased among ACSM during periods of Alpha and Delta coronavirus variant predominance, but not during the predominance of the Omicron variant. At the peak of the fill rate curve, in August 2021, rates were higher among men compared to women, older compared to younger age groups, senior officers compared to junior officers, senior enlisted compared to junior enlisted service members, and those with a bachelor’s or advanced degree compared to those without a bachelor’s degree. Ivermectin prescriptions were more likely to have been filled at a retail pharmacy than at a military hospital or clinic. During the COVID-19 pandemic fill rates for ivermectin prescriptions among ACSM increased, including those without a qualifying diagnosis. Rates peaked in August 2021 but subsequently declined. The decrease in ivermectin fill rates was coincident with vigorous efforts to correct previous misinformation and implement pre-authorization requirements for prescriptions. Research on the impact of unproven online claims about clinical and public health interventions has potential to curtail future unnecessary and potentially harmful treatments.

**What are the new findings?:**

Ivermectin prescription fill rates increased among active component service members early in the COVID-19 pandemic when misinformation about the effectiveness of ivermectin for prevention and treatment of COVID-19 was widespread.

**What is the impact on readiness and force health protection?:**

This study contributes to the understanding of ivermectin prescription uptake among U.S. military members during the early phases of the COVID-19 pandemic, a period of abundant online information purporting its benefit but insufficient evidence to support its use for COVID-19 prevention and treatment. This study supports the call by the U.S. Surgeon General to expand research that deepens our understanding of health misinformation, who is most susceptible, and which strategies are effective in addressing it.

## 1 BACKGROUND

Ivermectin, an anti-parasitic drug, was identified as a potential treatment for coronavirus disease 2019 (COVID-19) early in the pandemic. Numerous non-peer-reviewed publications touted the benefit of ivermectin, and it was heavily promoted online. [1] Many of these studies were subsequently found to have methodological flaws, and some were withdrawn because of data fraud—but not before their widespread circulation. A large study that purported ivermectin mortality reduction of up to 99% was viewed online more than 150,000 times, cited more than 30 times, and was included in several meta-analyses before it was retracted. [2,3,4]

Despite multiple studies that found insufficient evidence to support ivermectin use for COVID-19 prevention or treatment, [5,6,7,8] and alerts discouraging its use, [9,10] national ivermectin prescription monitoring showed that retail dispensing of ivermectin increased significantly during the COVID-19 pandemic. [11,12] Increased calls to U.S. poison control centers for adverse ivermectin reactions were also reported. On August 26, 2021, the U.S. Centers for Disease Control and Prevention (CDC) issued a Health Alert Network notice, “Rapid Increase in Ivermectin Prescriptions and Reports of Severe Illness Associated with Use of Products Containing Ivermectin to Prevent or Treat COVID-19,” that indicated a 24-fold increase in ivermectin dispensing from U.S. outpatient retail pharmacies compared to the pre-pandemic baseline. The CDC notice reminded prescribers that the U.S. Food and Drug Administration (FDA) did not approve ivermectin for COVID-19 prevention or treatment. [13] The following week, the American Medical Association, American Pharmacists Association, and American Society of Health-System Pharmacists published a statement strongly opposing ivermectin ordering, prescribing, and dispensing to prevent or treat COVID-19 outside a clinical trial. Many medical facilities and organizations including the U.S. Military Health System (MHS) instituted pre-authorization requirements for ivermectin prescriptions soon thereafter. This measure reinforced the Department of Defense (DOD)’s March 2021 COVID-19 Practice Management Guide recommending against use of ivermectin for treatment of COVID-19, except in a clinical trial. [14

Misinformation has various definitions in the medical literature. [15] This article uses the definition proposed by Johns Hopkins University: Medical misinformation is misleading information that is contrary to the best available evidence. [16] This definition recognizes that what qualifies as misinformation may change over time as new evidence emerges.

Medical misinformation is not a new phenomenon, but it has gained added significance since the dawn of the internet, which allows the spread of misinformation at unprecedented speed, and on an unparalleled scale. [17] The U.S. Surgeon General considers health misinformation a serious threat to public health due to its ability to cause confusion, promulgate mistrust, harm people’s health, and undermine public health efforts. [18] The DOD warns that adversaries are becoming more assertive in use of disinformation, which is defined as false or misleading information shared with malicious intent, in their attempts to sow distrust and disrupt world order. [19] These efforts target all sectors of government, including public health. [20] Examples include efforts by the former Soviet Union to attribute the HIV pandemic to U.S. government efforts to develop biological weapons, [21] and Russian internet troll activity between 2014 and 2017 that “weaponized” content about vaccines to fuel political and social discord. [22] During the COVID-19 pandemic, both Russia and China sponsored conspiracy narratives that included endorsement of ivermectin as an effective treatment for COVID-19, but which asserted that this was withheld from the public by a “Big Pharma cabal.” [23]

The MHS serves approximately 9.6 million beneficiaries including 1.4 million active duty service members. [24] In addition to promoting the health of its beneficiaries, the mission of the MHS is to ensure service members are prepared to defend the nation, and that uniformed medical personnel are trained to provide medical care in support of military operations.

The purpose of this study was to determine whether ivermectin prescriptions increased among active component service members (ACSM) during the COVID-19 pandemic, whether fill rates varied by subpopulation, and how fill rates changed over time as information that discouraged ivermectin use for COVID-19 prevention or treatment increased. This study supports the call by the U.S. Surgeon General to expand research that deepens our understanding of health misinformation, who is most susceptible, and which strategies are effective in addressing it. [18]

## 2 METHODS

This study was determined to qualify as Not Research by the DHA Director of the Office of Research Protections on February 7, 2022. The surveillance period was January 1, 2017 to March 31, 2022. The study population included all ACSM in the Army, Navy, Air Force, and Marine Corps who served at least 1 day on active duty during the surveillance period. All data used in this analysis were derived from records maintained in the Defense Medical Surveillance System (DMSS). These records document both ambulatory encounters and hospitalizations of ACSM of the U.S. Armed Forces in fixed military and civilian (if reimbursed through MHS) treatment facilities. In addition, DMSS contains data from the Pharmacy Data Transaction Service (PDTS), which includes dispensed outpatient prescriptions for service members at military hospitals and clinics, as well as civilian purchased care.

To identify dispensed outpatient ivermectin prescriptions, records where the drug name included “IVERMECTIN” or “STROMECTOL” were identified in DMSS. Only prescriptions for oral tablets were included; ointments and creams were excluded. Rates of dispensed oral ivermectin prescriptions were calculated as the number of prescriptions per 100,000 person-years (p-years) and results were stratified by demographic characteristics.

To determine the rate of ivermectin prescriptions among those without an ivermectin-qualifying diagnosis (e.g., helminthiasis, lice, scabies), inpatient and outpatient records that contained a diagnosis for any International Classification of Diseases, 10th Revision (ICD-10) code listed in **Table 1**, in any diagnostic position, were extracted from DMSS. Service members were considered to have an ivermectin-qualifying diagnosis if the diagnosis occurred within 90 days preceding the ivermectin prescription. A service member was considered to have a prior diagnosis of COVID-19 if a medical record of ICD-10 code U07.1 in any diagnostic position during an inpatient or outpatient encounter, a positive PCR or antigen test, or a reportable medical event (RME) for COVID-19 were documented on or before the date of the ivermectin prescription. Data from laboratory test results and RMEs were derived from the Armed Forces Health Surveillance Division (AFHSD) “master positive list” of COVID-19 cases, which consolidates COVID-19 cases based on diagnosis, laboratory results, and RMEs, and has been used by AFHSD to track COVID-19 cases among MHS beneficiaries since the beginning of the COVID-19 pandemic.

## 3 RESULTS

**Figure 1:**
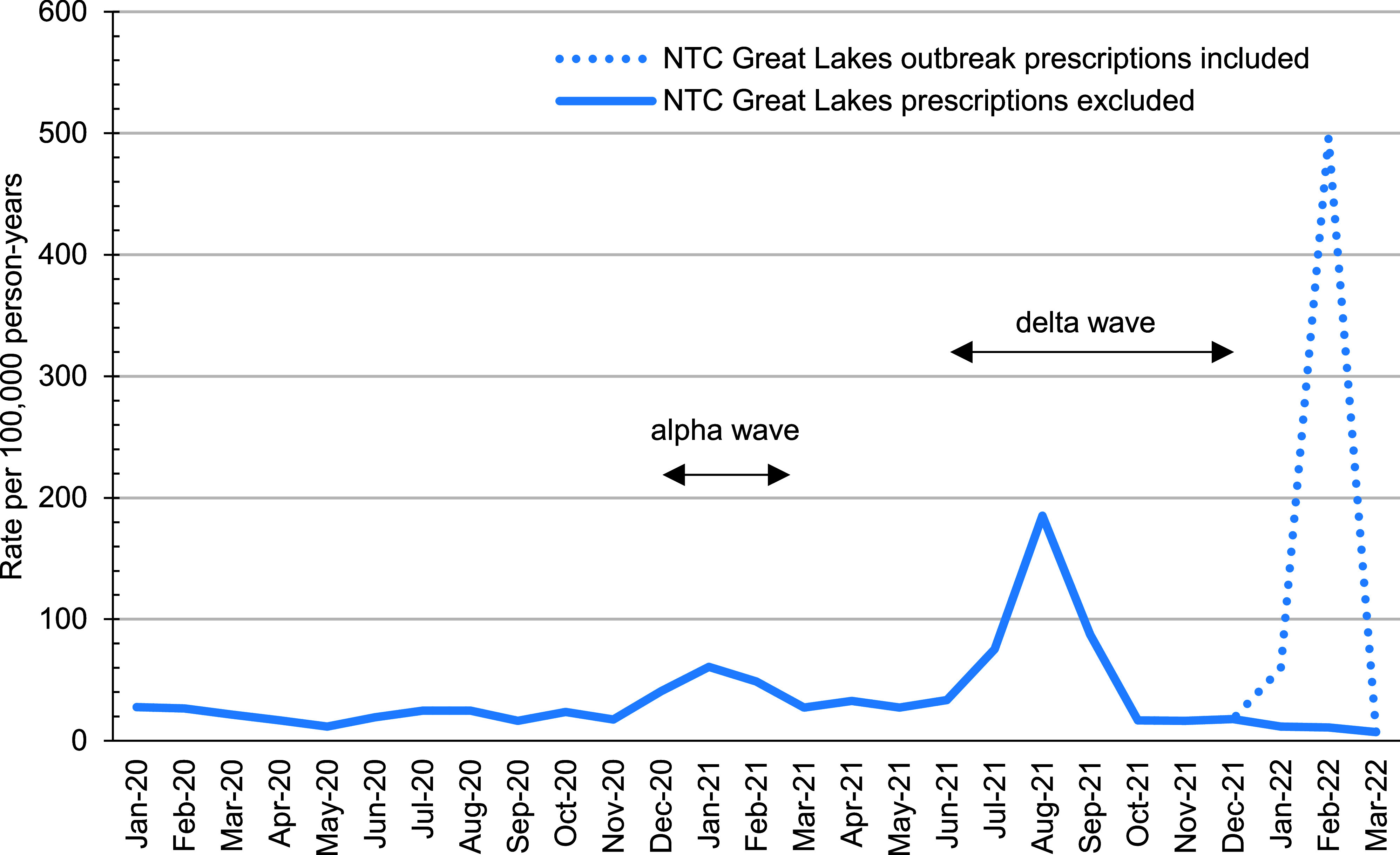
Monthly Rate of Dispensed Outpatient Oral Ivermectin Prescriptions, Active Component, U.S. Armed Forces, January 2020-March 2022

The annual rate of dispensed prescriptions was stable from calendar years 2017 through 2020, at 25.6 prescriptions per 100,000 p-years in 2017, 22.7 in 2018, 27.2 in 2019, and 22.7 in 2020 (data not shown). In 2021 the annual prescription rate more than doubled: to 52.8 per 100,000 p-years. Ivermectin prescription rates peaked in August 2021, during the period of Delta variant predominance, at 185.3 per 100,000 p-years, then declined through the end of 2021 (**Figure 1**).

A large peak in ivermectin prescriptions between January 2022 (59.9 per 100,000 p-yrs) and February 2022 (496.4 per 100,000 p-yrs) was driven by Navy service members receiving prescriptions at Naval Training Center (NTC) Great Lakes. Communication with the NTC Great Lakes Public Health Emergency Officer revealed a scabies outbreak among approximately 500 recruits during this period, and the entire recruit population had been prophylactically treated with oral ivermectin. These data are presented in the dotted line in **Figure 1**. Prescriptions filled at NTC Great Lakes during January and February 2022 were excluded from further analysis due to their identified outbreak-related purpose. After the NTC Great Lakes prescriptions were removed, a total of 2,018 oral ivermectin prescriptions were dispensed among 1,656 individuals between January 1, 2017 and March 31, 2022 (**Table 2**). The prescription rate declined sharply after August 2021 and remained low and steady from October 2021 until the end of the study period. A total of 1,400 individuals had only 1 oral ivermectin prescription between January 2017 and March 2022, while 256 individuals had 2 or more (data not shown).

Between January 2017 and March 2022, most prescriptions for ivermectin were filled at military hospitals or clinics, compared to mail order, retail, in-theater, and Veterans Administration pharmacies. Prescription rates from 2017 until March 2022 were similar between men and women (**Table 2**). Prescription rates increased steadily with increasing age, from 12.8 per 100,000 p-years among service members less than 20 years old, to 66.8 per 100,000 p-years among service members 45 years and older. Rates were slightly higher in the Air Force, followed by the Navy, Army, and Marine Corps. Rates were highest among non-Hispanic White Service members and lowest among non-Hispanic Black Service members. Rates were also higher among senior officers compared to senior enlisted, junior officers, and junior enlisted service members. Compared to those in other military occupations, rates were highest for health care personnel, followed by pilot/air crew. Rates were highest in the South and Midwest compared to other regions of the U.S.

To compare patterns in ivermectin prescription rates prior to the COVID-19 pandemic with rate patterns at the height of the Delta wave, rates from January 2017 to December 2019 and August 2021 were examined separately. Several differences were noted (**Table 2**). In August 2021, most prescriptions were filled at a retail pharmacy (165.1 per 100,000 p-yrs compared to 17.6 per 100,000 p-yrs filled at a military hospital or clinic). Between January 2017 and December 2019 the prescription fill rate was 8.4 per 100,000 p-years at retail pharmacies compared to 16.1 per 100,000 p-years at military hospitals or clinics. In August 2021, men had a higher ivermectin prescription fill rate (197.6 per 100,000 p-yrs) compared to women (126.6 per 100,000 p-yrs), whereas rates were similar between men and women, 25.1 and 25.6 per 100,000 p-years, respectively, from January 2017 to December 2019.

In both August 2021 and the period preceding 2020, the ivermectin prescription rate increased with increasing age, but the pattern was more marked in August 2021. The August 2021 rate among those older than 45 years was 640.7 per 100,000 p-years—the rate among those younger than 20 years was 36.2 per 100,000 p-years. During January 2017 through December 2019 period, the rate among those older than 45 years was 49.3 per 100,000 p-years, while the rate among those younger than 20 years was 13.5 per 100,000 p-years.

Warrant officers had much higher prescription fill rates than senior or junior officers in August 2021 (685.7 per 100,000 p-yrs among warrant officers vs. 83.7 per 100-000 p-yrs in junior officers, and 225.6 per 100-000 p-yrs among senior officers). Senior enlisted ACSM had a higher rate from January 2017 through December 2019 (25.4 per 100,000 p-yrs), while warrant officers had the lowest fill rate (16.4 per 100,000 p-yrs). Among those for whom educational attainment is known, ivermectin prescription fill rates increased with higher educational levels in both August 2021 and the January 2017 through December 2019 period: 300.5 per 100-000 p-years among those with a bachelor’s degree or an advanced degree compared to 127.3 per 100-000 p-years among those with a high school diploma or less in August 2021; 37.1 per 100-000 p-years among those with a bachelor’s degree or an advanced degree compared to 19.7 per 100-000 p-years among those with a high school diploma or less in the prior period.

**Table 2** includes additional information related to comparative rates based on race, service affiliation, military occupational specialty, and region of assignment.

**Figure 2:**
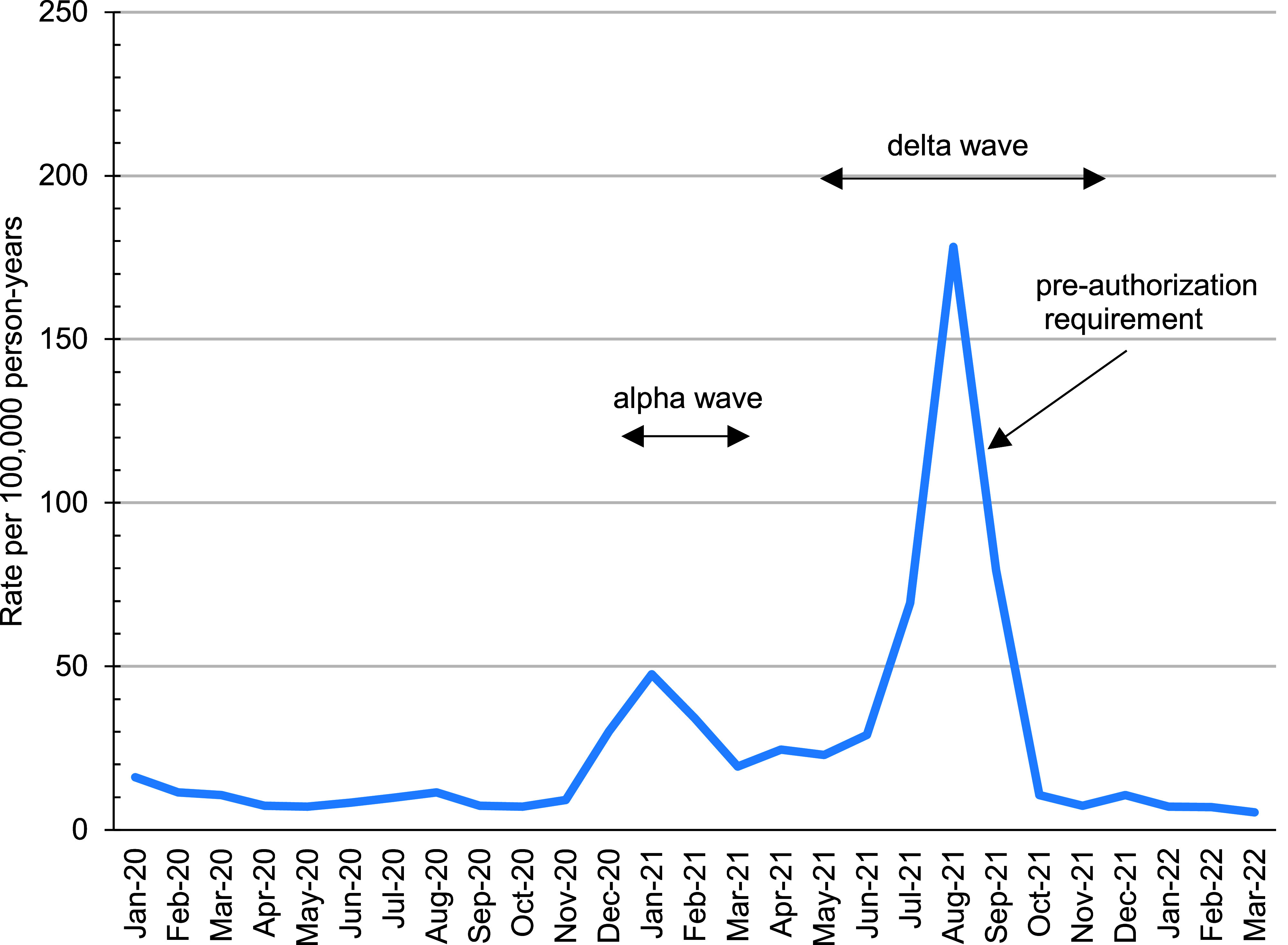
Incidence of Eosinophilic Esophagitis in Active Component Service Members by Sex, 2009-2021 Abbreviation: P-yrs, person-years.

Nearly two-thirds (n=1,308, 64.8%) of the 2,018 ivermectin prescriptions dispensed during the January 2017 through March 2022 surveillance period occurred among individuals without a qualifying ivermectin diagnosis within the 90 days preceding their ivermectin prescription (data not shown). The annual rate of ivermectin prescriptions without a qualifying diagnosis remained relatively stable between 2017 and 2020 but nearly quadrupled in 2021, from 11.4 per 100,000 p-years in 2020 to 44.7 per 100,000 p-years (data not shown). The monthly prescription rate peaked in January 2021 (i.e., Alpha wave) at 47.6 per 100,000 p-years and then peaked at the highest rate observed during the surveillance period in August 2021 (i.e., Delta wave), at 178.3 per 100,000 p-years (**Figure 2**).

Among the 2,018 ivermectin prescriptions dispensed during the study period, 978 were dispensed following the declaration of the COVID-19 pandemic in March 2020 by the World Health Organization (data not shown). Among those 978 prescriptions, 324 (33%) were filled for individuals with a prior diagnosis of COVID-19. A higher proportion of prescriptions with a prior COVID-19 diagnosis were dispensed to those who did not have a qualifying ivermectin diagnosis (295/739=40%) compared to those with a qualifying ivermectin diagnosis (29/239=12%).

## 4 DISCUSSION

This study revealed increased ivermectin prescription fill rates among U.S. ACSM during coronavirus Alpha and Delta variant waves, including increased use among those without a qualifying diagnosis. The highest ivermectin fill rates among U.S. ACSM occurred during the period of Delta variant predominance in the U.S., from July 2021 through September 2021. During this period, there was a 7.3-fold increase in prescription fill rates compared to the baseline period (January 2017–December 2019), which correlates with the highest rate of U.S. online interest in ivermectin recorded by Google Trends. [25] During the week that ended on August 13, 2021, there was a 24-fold increase in ivermectin prescrition fills in the U.S. compared to the U.S. baseline. The second-highest ivermectin fill rates occurred from December 2020 until early March 2021, during the Alpha variant wave, when online interest in ivermectin also increased above baseline. Despite online interest, ivermectin prescription fill rates did not increase during Omicron variant predominance, when daily COVID-19 case rates reached the highest level recorded in the U.S. [25]

The reason ivermectin prescription fill rates did not increase among ACSM during the Omicron wave is likely multifactorial. Retractions of invalid early studies, along with increased availability of evidence demonstrating lack of ivermectin efficacy against COVID-19, as well as vigorous efforts by private and governmental organizations to call attention to false claims and risks associated with off-label use of ivermectin likely contributed. [4,5,6,7,8] It is also possible the requirement for prescription pre-authorization, implemented by the military after the Delta wave and prior to Omicron, played a role. A significant proportion of ivermectin prescriptions were filled at retail pharmacies in August 2021—while the reverse was true from January 2017 to December 2019—making the impact of the pre-authorization requirement unclear. In addition, this analysis did not evaluate those providers who wrote prescriptions for ivermectin. It is unclear what proportion of prescriptions were provided by providers within the MHS and what proportion were written by civilian providers outside the MHS.

During the peak of the Delta variant wave, in August 2021, comparatively higher rates of ivermectin prescription fills were seen among men compared to women; by comparison, rates according to sex were similar from January 2017 to December 2019. Rates of ivermectin prescription fills were also much higher among older than younger service members in August 2021, whereas the difference in fill rates by age was less marked prior to August 2021. Given that rank and education typically increase with age, it is not surprising that warrant officers had significantly higher fill rates than junior and senior enlisted service members, and those with a bachelor’s or advanced degree had significantly higher fill rates in August 2021 than those with less formal education, compared to previous years.

The findings related to education and ivermectin fill rates are interesting, given that the groups with higher levels of education are traditionally considered less susceptible to medical misinformation. In particular, Scherer et al. found that less educational attainment was consistently associated with greater misinformation susceptibility. [26] Pan et al. also found that increasing education, as well as age, were protective against acceptance of misinformation. [27] Interestingly, data presented here are consistent with more recent data reported by Perlis et al., who also found that men, those with a college degree (compared to less education), and those among the highest age group, compared to younger individuals, were all more likely to use non-evidence-based treatments during the COVID-19 pandemic. [28]

A possible explanation for these findings is that older, more senior, and higher-educated individuals have a greater sense of self-efficacy when interpreting online information, and were more proactive in their requests for ivermectin during interactions with health care providers. While it may be assumed that greater educational attainment is protective against misinformation, this may not be true. Studies show that misinformation can go unrecognized by consumers, regardless of educational status, and that even short exposures to misinformation or disinformation can significantly affect unconscious behavior. [29]

The relatively higher fill rates in August 2021 among non-Hispanic Whites (compared to other racial categories), among airmen compared to other ACSM, pilots compared to other military occupations, and those living in the South, compared to other regions, is unclear. Further stratification of these groups and additional studies could offer insight into these findings.

Medical misinformation has resulted in significant insurance subsidization of ineffective care, [30] despite the Federation of State Medical Boards’ efforts to discipline practitioners who spread misinformation and disinformation related to COVID-19 management. [31] Direct-to-consumer advertising of prescription drugs, for-profit interventions, and patient reliance on online medical information are expected to increase. [32] These trends have the potential to affirmatively influence patients’ use of media-based medicine, and dissuade their use of evidence-based medicine.

State-sponsored online disinformation, including that targeting public health, has increased in recent years. [33] The availability of social media, increasingly sophisticated algorithms, and rapidly evolving artificial intelligence all increase capacity for conflict escalation within the digital realm that can undermine evidence-based public health responses. [34] These efforts can directly discredit credible interventions, such as efficacious vaccines, as well as indirectly sow mistrust and delegitimize public health and other governmental institutions.

Potential population-level and organizational countermeasures against misinformation and disinformation include debunking and pre-bunking, [35] increased investment in research on misinformation, [18] and modernization of public health communications, including implementation of infodemic surveillance systems. [36] The National Strategy for the COVID-19 Response and Pandemic Preparedness outlines the federal government’s commitment to mitigating misinformation and disinformation by ensuring Americans have access to science-based information, and developing capacities for quickly identifying disinformation and misinformation. [37] While recognizing constitutional concerns related to free speech, [38] Johns Hopkins University Bloomberg School of Public Health calls for expansion of the federal strategy, including improved resources for public verification of questionable content and increased coordination among constituencies to establish a multiagency national security response effort prioritizing management of public health disinformation from both domestic and international sources. [37] The urgency of managing misinformation and disinformation is highlighted in DOD’s Strategy for Operations in the Information Environment. [19] While this document does not specifically address health-related misinformation and disinformation, the findings here suggest that it should.

This report is subject to several limitations. First, this is a descriptive study of a small population, and conclusions based on these findings require further validation. Second, prescriptions filled outside the MHS and not reimbursed through TRICARE, as well as those obtained without a prescription, were not captured in this analysis. Third, prescription fill rates do not necessarily equate to prescription use; fill rates may both under- and overestimate actual drug use. Fourth, qualifying diagnoses were based on encounter data only. Inclusion of laboratory or other diagnostic data to identify qualifying diagnosis may have increased the number of individuals with a qualifying diagnosis. Fifth, the 90-day period for qualifying ivermectin-qualifying diagnoses was meant to identify as many individuals as possible and is somewhat arbitrary. Shortening or lengthening this period could potentially decrease or increase, respectively, individuals with a qualifying diagnosis. Finally, Google Trends is not a measure of increased exposure to misinformation or disinformation, and any suggestion of a relationship between ivermectin fill rates and misinformation or disinformation herein should be considered exploratory.

As with their civilian counterparts, U.S. military members’ ivermectin prescription fill rates increased during the early phases of the COVID-19 pandemic. This trend was coincident with significant online information espousing ivermectin benefits, but during a period when there was lack of scientific evidence to support its use and no FDA approval of ivermectin for COVID-19 prevention or treatment. Older and more educated individuals had relatively high prescription fill rates, counter to the assumption that age and education protect against online misinformation.

Misinformation and disinformation have assumed increasing significance in the digital information age, with direct relevance to both pandemic preparedness and military operations. Bernard et al. suggest we are entering a new era of biowarfare, one that relies less on a biological weapon and more on the ability to weaponize natural outbreaks, with the goal of destabilizing social, political, and economic systems. [33] Understanding how medical misinformation and disinformation affect the military and how these impacts vary among and within subpopulations is important for ensuring the health of military members as well as national security. [18]

## Figures and Tables

**Table 1:** Ivermectin-qualifying Diagnoses

**Diagnosis**	**ICD-10-CM code**
Onchocerciasis	B73^*^
Strongyloidiasis	B78^*^
Ascariasis	B77^*^
Gnathostomiasis	B83.1
Hookworm-related cutaneous larva migrans	B76.8, B76.9
Lice (pediculosis and phthiriasis)	B85^*^
Mansonelliasis	B74.4
Scabies	B86
Trichuriasis	B79

Abbreviation: ICD-10-CM, International Classification of Diseases, 10th Revision, Clinical Modification.

^*^Indicates all subsequent digits/characters are included.

**Table 2:** Rate of Dispensed Outpatient Oral Ivermectin Prescriptions per 100,000 Person-Years, Active Component, U.S. Armed Forces, January 2017–March 2022

	**Jan 2017-Mar 2022**	**Jan 2017-Dec 2019** (prior to start of COVID-19 pandemic)	**Aug 2021** (peak of ivermectin dispensation)
	**No.**	**Rate**	**No.**	**Rate**	**No.**	**Rate**
**Total**	2,018	29.3	981	25.2	211	185.3
**Prescription category**						
Mail order	8	0.1	2	0.1	3	2.6
Military hospital or clinic	1,026	14.9	628	16.1	20	17.6
Retail	960	14.0	329	8.4	188	165.1
In-Theater	20	0.3	18	0.5	0	0.0
VA	4	0.1	4	0.1	0	0.0
**Sex**						
Male	1,710	29.9	817	25.1	186	197.6
Female	308	26.6	164	25.6	25	126.6
**Age, years**						
under 20	66	12.8	41	13.5	3	36.2
20-24	442	20.0	247	19.8	34	93.1
25-29	391	24.6	190	21.2	38	144.0
30-34	392	35.9	198	32.0	41	227.2
35-39	341	42.2	136	30.2	45	329.7
40-44	217	52.8	97	42.1	24	346.0
45+	169	66.8	72	49.3	26	640.7
**Service**						
Army	709	28.6	317	22.6	85	206.4
Navy	533	30.7	291	29.9	41	139.9
Air Force	559	32.8	269	27.9	68	241.5
Marine Corps	217	22.6	104	18.7	17	111.7
**Race and ethnicity**						
Non-Hispanic White	1,287	33.6	644	29.4	137	219.9
Non-Hispanic Black	174	15.7	75	11.9	19	103.6
Hispanic	297	26.0	142	22.8	27	134.0
Other/unknown	260	32.7	120	26.6	28	213.8
**Rank**						
Junior enlisted (E1-E4)	585	19.7	316	18.7	41	83.7
Senior enlisted (E5-E9)	837	31.0	386	25.4	101	225.6
Warrant officer (WO1-WO5)	37	38.3	9	16.4	11	685.7
Junior officer (O1-O3)	263	38.5	127	32.8	29	256.3
Senior officer (O4-O10)	296	67.7	143	57.9	29	401.6
**Marital status**						
Single, never married	705	23.4	387	23.1	42	81.6
Married	1,184	33.6	535	26.4	159	280.1
Other/unknown	129	37.8	59	30.8	10	176.2
**Education level**						
High school or less	967	22.2	485	19.7	92	127.3
Some college	302	35.5	146	29.6	33	246.2
Bachelor's or advanced degree	686	44.7	318	37.1	78	300.5
Other/unknown	63	47.0	32	41.7	8	353.4
**Military occupation**						
Combat-specific	284	30.1	136	25.6	32	203.1
Motor transport	48	23.4	24	21.0	5	141.9
Pilot/air crew	93	38.0	43	30.8	16	398.7
Repair/engineering	526	25.8	251	21.8	59	173.1
Communications/intelligence	471	32.1	213	25.7	54	221.5
Health care	260	43.7	152	44.5	14	147.3
Other/unknown	336	24.2	162	20.5	31	137.1
**Geographic region**						
Northeast	45	22.7	23	20.3	7	214.8
Midwest	149	33.5	89	35.4	12	161.1
South	1,179	37.3	520	29.1	145	277.2
West	387	21.9	205	20.6	31	105.2
Overseas	160	20.9	93	21.5	10	78.8
Unknown/missing	98	18.1	51	16.1	6	68.8

Abbreviation: VA, Veterans Administration.

## AUTHOR AFFILIATIONS

Uniformed Services University of the Health Sciences: CAPT Clausen; Epidemiology and Analysis Branch, Armed Forces Health Surveillance Division, Defense Health Agency: Ms. Murray, Dr. Stahlman

## DISCLAIMERS

The opinions and assertions expressed herein are those of the authors and do not reflect the official policy nor position of the Uniformed Services University of the Health Sciences, the Department of Defense, or the U.S. Government.

References to non-Federal entities or products do not constitute nor imply a Department of Defense or Uniformed Services University of the Health Sciences endorsement.

This work was prepared by a military or civilian employee of the U.S. Government as part of that individual’s official duties and therefore is in the public domain and does not possess copyright protection. Public domain information may be freely distributed and copied; however, as a courtesy it is requested that the Uniformed Services University and the author be given an appropriate acknowledgment.
